# Graphene–Multiwalled Carbon Nanotubes Modified Glassy Carbon Electrodes for Simultaneous Detection of Ascorbic Acid, Dopamine, and Uric Acid

**DOI:** 10.1021/acsomega.4c09646

**Published:** 2025-02-20

**Authors:** Hsien-Hsu Hsieh, Jie-Yu Xu, Jing-Tong Lin, Yun-Ting Chiang, Yu-Ching Weng

**Affiliations:** † Department of Pathology and Laboratory Medicine, Taichung Veterans General Hospital, Taichung 407219, Taiwan; ‡ Department of Chemical Engineering, Feng Chia University, Taichung 407102, Taiwan

## Abstract

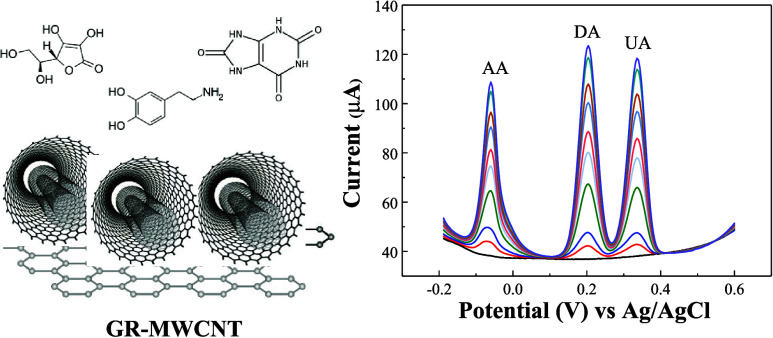

This study compares the sensing performance of glassy carbon electrodes (GCE) modified with graphene (GR), multiwalled carbon nanotubes (MWCNTs), and graphene–multiwalled carbon nanotube (GR-MWCNT) composites for the simultaneous detection of ascorbic acid (AA), dopamine (DA), and uric acid (UA). Among these electrodes, GR-MWCNT/GCE exhibited the highest sensitivity and the lowest detection limit. Using differential pulse voltammetry (DPV), AA, DA, and UA can be simultaneously detected at low potentials of −0.032, 0.206, and 0.34 V vs Ag/AgCl, with sensitivities of 0.076, 1.38, and 0.181 μA μM^–1^ and detection limits (LOD) of 6.71, 0.58, and 7.30 μM, respectively. The GR-MWCNT/GCE also demonstrated good stability, reproducibility, and excellent anti-interference capability. This newly fabricated sensor was confirmed to be applicable for the simultaneous detection of AA, DA, and UA in real serum and urine samples.

## 1 Introduction

Ascorbic acid (AA) is a natural antioxidant that reacts with free radicals, promoting wound healing, preventing infections, and delaying aging. However, excessive intake may irritate the gastrointestinal mucosa, leading to nausea, vomiting, upper abdominal pain, and diarrhea.^1^ On the other hand, insufficient intake can result in fatigue, muscle weakness, swollen and bleeding gums, increased susceptibility to infections, and delayed wound healing. Dopamine (DA) is a critical neurotransmitter. Excess dopamine may cause heart failure, tachycardia, hypertension, and drug addiction, while insufficient secretion can lead to stress, depression, Parkinson’s disease, schizophrenia, and Alzheimer’s disease.^2−4^ Uric acid (UA) is the final product of purine metabolism. When UA levels exceed 7 mg/dL, sodium urate crystals may accumulate in the joints, causing gouty tophi and related complications, such as hypertension and diabetes.^5^ Thus, detecting the concentrations of AA, DA, and UA in the body is crucial. Rapid detection methods are essential for monitoring and preventing these diseases.

There are various methods for detecting AA, DA, and UA, including spectrophotometry,^6^ fluorescence spectrophotometry,^7,8^ reverse-phase high-performance liquid chromatography (HPLC),^9^ 2,4-dinitrophenylhydrazine colorimetry,^10^ gas chromatography–mass spectrometry (GC-MS),^11^ colorimetry,^12,13^ and enzymatic methods.^14^ However, most of these methods involve tedious experimental processes, relatively difficult sample preparation, longer analysis times, and lower selectivity and sensitivity. In contrast, electrochemical methods offer advantages such as rapid response, higher sensitivity and selectivity, simple equipment, low cost, and the potential for miniaturization.^15^ Nevertheless, simultaneously detecting AA, DA, and UA using electrochemical methods is challenging because their oxidation potentials are similar on bare electrodes, leading to signal overlap. Therefore, electrode modification is necessary to enhance selectivity, sensitivity, and lower detection limits.^15^ Modified electrodes can improve electrocatalytic activity for the three analytes.^2,3^ The surface materials of modified electrodes can be combinations of nanomaterials, conductive polymers, composite materials, inorganic compounds, and ionic liquids.^16−24^

Multiwalled carbon nanotubes (MWCNTs) have attracted widespread attention due to their large specific surface area, excellent chemical and mechanical properties, and high catalytic activity. Nevertheless, the strong van der Waals force interactions among MWCNTs lead to significant agglomeration, which limits their conductive properties.^24^ Thus, an effective strategy is required to harness the potential of MWCNTs. Graphene (GR), a two-dimensional carbon-based nanostructure with a honeycomb lattice, has attracted interest due to its high electrical conductivity, large surface area, exceptional chemical stability, and unique electronic and mechanical properties. The superior electrical conductivity of GR is utilized to reduce the resistance caused by MWCNT agglomeration. The synergistic interaction between the GR and MWCNTs is expected to enhance their electrocatalytic activity. Numerous studies have reported carbon-based or carbon-composite materials, such as graphene quantum dots (GQDs) with ionic liquid (IL),^16^ poly(3,4-ethylenedioxythiophene) (PEDOT)/rGO,^17^ porous nitrogen-doped graphene aerogels (HNGA),^18^ cationic polyfluorinated azobenzene/rGO,^19^ nitrogen-doped reduced graphene oxide (N-rGO),^20^ polymerized dianix yellow (PDY) with MWCNTs,^21^ carbon black (CB) and CNT codoped polyimide (PI),^22^ nitrogen-doped CNT arrays,^23^ gC_3_N_4_/MWNTs/GO,^24^ CNT-carbon fiber,^25^ gC_3_N_4_/GO,^26^ and Co, Mo/CNFs^27^ modified electrodes for detecting AA, DA, and UA. However, limited research has focused on GR-MWCNT composites for glassy carbon electrode (GCE) modification to simultaneously detect UA, AA, and DA. In this work, we present an effective strategy incorporating GR to mitigate the resistance caused by MWCNT agglomeration while simultaneously enhancing the electroactive surface area. This method harnesses the distinctive properties of GR to significantly enhance the catalytic performance of the GR-MWCNT composites, thereby advancing their application in electrochemical sensing.

## 2 Experimental Section

### 2.1 Materials and Reagents

Analytical grade dopamine (99%, Thermo Fisher Scientific), ascorbic acid (99%, Tokyo Chemical Industry), uric acid (99%, Sigma-Aldrich), graphene powder (Taiwan Carbon Materials), multiwalled carbon nanotube powder (Taiwan Carbon Materials), C_3_H_7_NO (99.5%, Showa), KH_2_PO_4_ (99.5%, Showa), Na_2_HPO_4_ (99%, J. T. Baker), NaCl (99%, Sigma-Aldrich), and KCl (99.5%, Showa) were used without further purification. All aqueous solutions were freshly prepared by using deionized water.

### 2.2 Preparation of GR/GCE, MWCNT/GCE, and GR-MWCNT/GCE

First, the GCE was polished with alumina powder, immersed in deionized water, and sonicated for 30 min using an ultrasonic cleaner to obtain a clean electrode. Then, 0.5 wt % of graphene powder was added to dimethylformamide (DMF) and stirred for 30 min with a magnetic stirrer, followed by 30 min of sonication to prepare a uniform graphene suspension. Next, 2 μL of the suspension was dropped onto the clean GCE and placed in an oven for 5 min, yielding the GR/GCE. Similarly, 0.5 wt % of MWCNT powder was added to DMF, stirred for 30 min, and sonicated for another 30 min to prepare a uniform MWCNT suspension. Afterward, 2 μL of the MWCNT suspension was dropped onto the GCE and baked for 5 min, resulting in the MWCNT/GCE. For the GR-MWCNT/GCE preparation, 0.25 wt % of graphene and 0.25 wt % of MWCNT powders were combined in DMF, stirred for 30 min, and sonicated for 30 min to create a uniform graphene-MWCNT suspension. Finally, 2 μL of this suspension was applied to the GCE and heated for 5 min, producing the GR-MWCNT/GCE.

### 2.3 Electrochemical Measurements and Characteristics of Electrodes

All electrochemical experiments were conducted at room temperature using a CHI Model 920D system. The GR/GCE, MWCNT/GCE, and GR-MWCNT/GCE served as the working electrodes, while the Ag/AgCl electrode and a platinum plate were used as the reference and counter electrodes, respectively. Cyclic voltammograms (CVs) were recorded in 0.1 M phosphate-buffered saline (PBS), both in the absence and presence of AA, DA, and UA. Due to its high detection sensitivity and low detection limit, differential pulse voltammetry (DPV) was also employed for the simultaneous detection of AA, DA, and UA. The scan potential window ranged from −0.2 to 0.6 V (vs Ag/AgCl saturated with KCl), with a pulse amplitude of 0.05 V, a pulse width of 0.1 s, and a pulse period of 0.2 s. The Raman spectra were recorded by a high-resolution Raman spectrometer (Andor BWII RAMaker SR-750, NEWTON). Transmission electron microscopy (HRTEM) measurement was performed on a HITACHI H-8100 electron microscope (Hitachi, Tokyo, Japan) with an accelerating voltage of 200 kV.

## 3 Results and Discussion

### 3.1 Characterization of GR, MWCNT, and GR-MWCNT Composites

**Figure 1:**
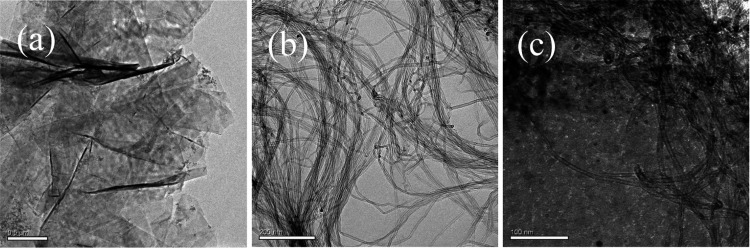
HRTEM images of the (a) GR, (b) MWCNT, and (c) GR-MWCNT composites.

The surface morphologies of GR, MWCNT, and GR-MWCNT composites were observed by using HRTEM, as shown in Figure 1a–c, respectively. The graphene morphology consists of flat sheets with noticeable wrinkles, while the MWCNTs appear as tubular structures with an average inner diameter of approximately 7 nm. Figure 1c illustrates the random distribution of MWCNTs across the graphene sheets.

**Figure 2:**
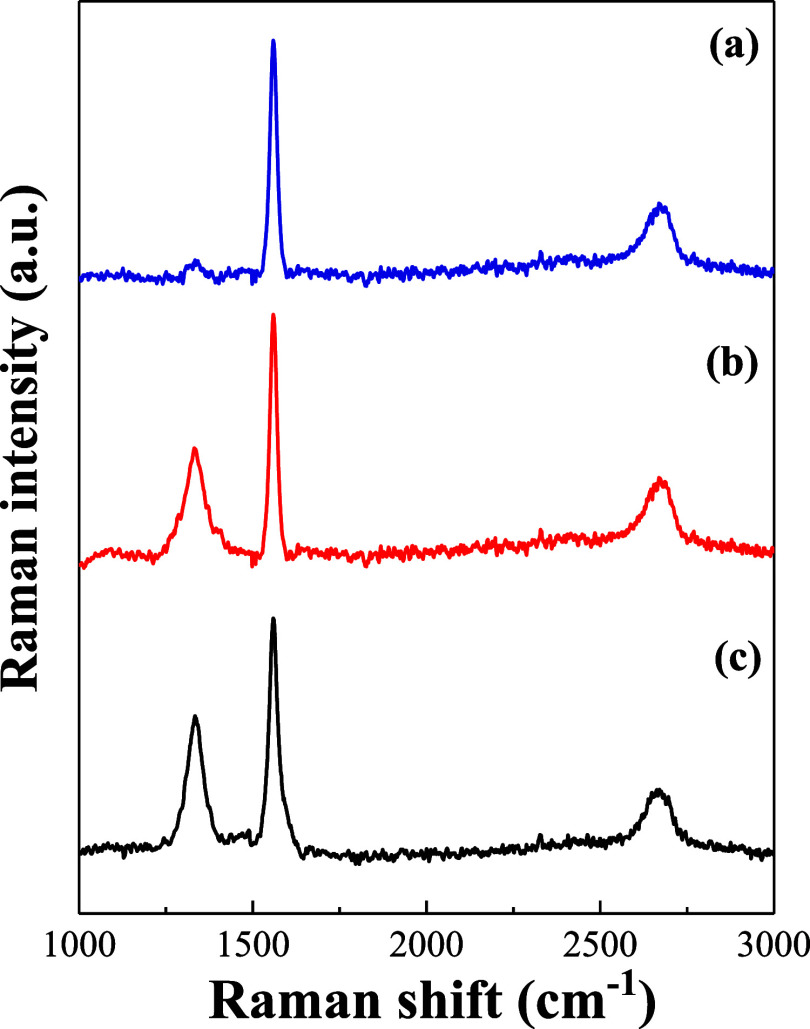
Raman spectra of the (a) GR, (b) MWCNT, and (c) GR-MWCNT composites.

Figure 2 shows the Raman spectra of the GR, MWCNT, and GR-MWCNT composites. The G peak (1580 cm^–1^) and D peak (1350 cm^–1^) correspond to the tangential atomic vibrations of the graphite lattice and various defect states in the graphite lattice, respectively. The degree of defects can also be determined by the intensity ratio of the D peak to the G peak (*I*_D_/*I*_G_). A higher *I*_D_/*I*_G_ ratio indicates more defects. The *I*_D_/*I*_G_ values for GR, MWCNT, and GR-MWCNT composites are 0.26, 0.56, and 0.70, respectively. This indicates that the GR-MWCNT composites have the highest degree of defects among the three materials. A highly defective electrode surface is advantageous for the simultaneous detection of AA, DA, and UA.

### 3.2 Electrochemical Active Surface Area (ECSA) of GR/GCE, MWCNT/GCE, and GR-MWCNT/GCE

**Figure 3:**
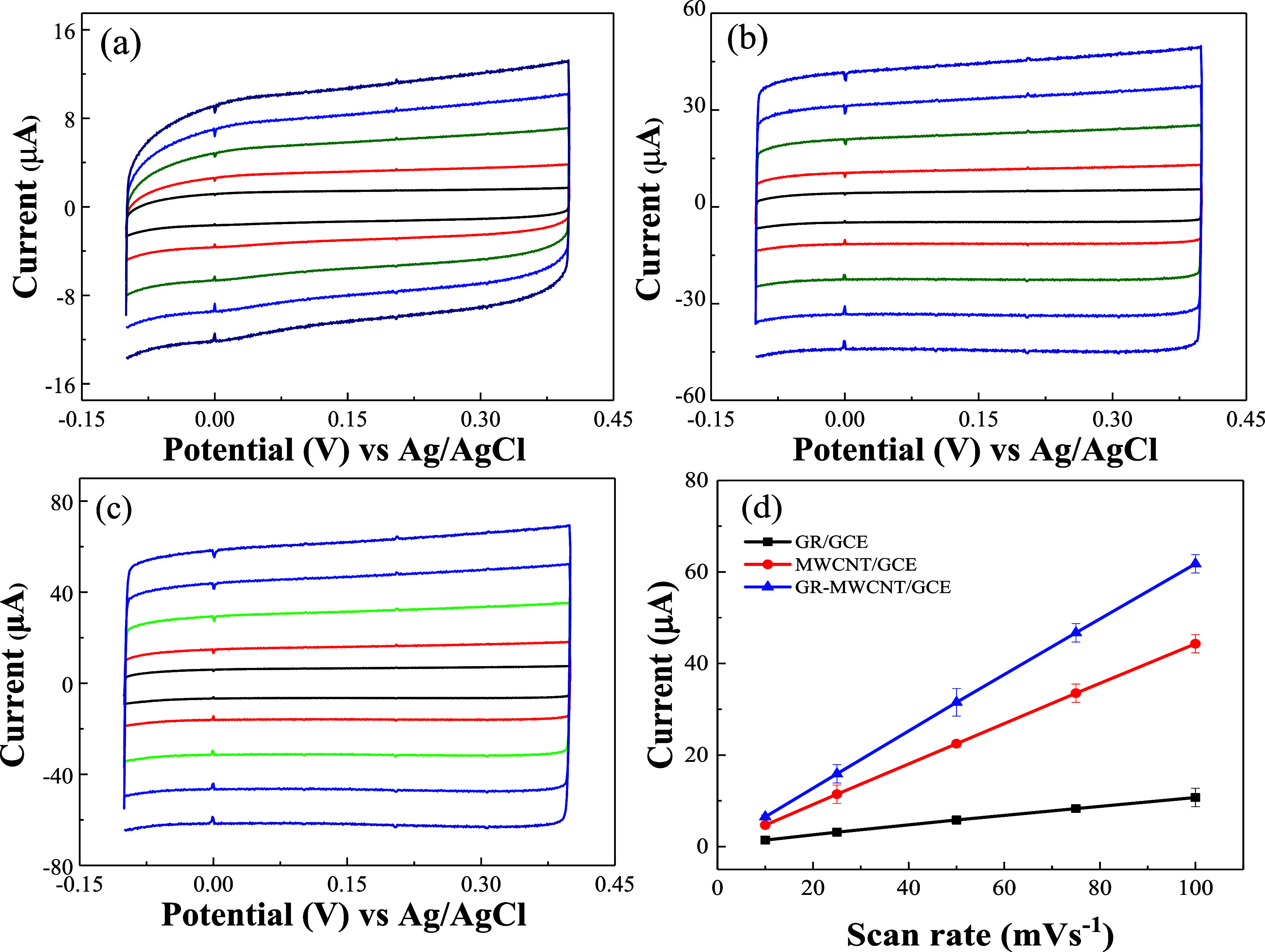
Electrochemical active surface area characterizations for the (a) GR, (b) MWCNT, and (c) GR-MWCNT electrodes and (d) linear fitting curves of scan rate vs difference in double-layer charging currents of the GR, MWCNT, and GR-MWCNT electrodes.

ECSA of GR/GCE, MWCNT/GCE, and GR-MWCNT/GCE was evaluated using the electrochemical double-layer capacitance (*C*_DL_). ECSA of the electrodes is directly proportional to the electrochemical double-layer capacitance (*C*_DL_),^28^ as shown in eq 1. Here, Cs represents the specific capacitance of the catalyst. The general specific capacitance of *C*_s_ (0.035 mF/cm^2^) was used. The electrochemical double-layer capacitance of the electrodes can be determined from the slope of the linear relationship between the non-faradic current and the scan rate, as described in eq 2. Figure 3a–c shows the cyclic voltammetry scans of GR/GCE, MWCNT/GCE, and GR-MWCNT/GCE, respectively, in a 0.1 M PBS solution, within a potential window of −0.1 to 0.4 V vs Ag/AgCl, with varying scan rates. By plotting the charging current (*i*_c_) against the scan rate (*v*), we obtained the slope corresponding to the electrochemical double-layer capacitance (*C*_DL_), as illustrated in Figure 3d.
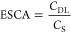
1
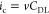
2ECSA of GR/GCE, MWCNT/GCE, and GR-MWCNT/GCE are 0.0385, 0.0803, and 0.112 cm^2^, respectively, which are 1.38, 2.87, and 4 times that of the GCE.

### 3.3 Electrochemical Reactivity and the Effect of Solution pH

**Figure 4:**
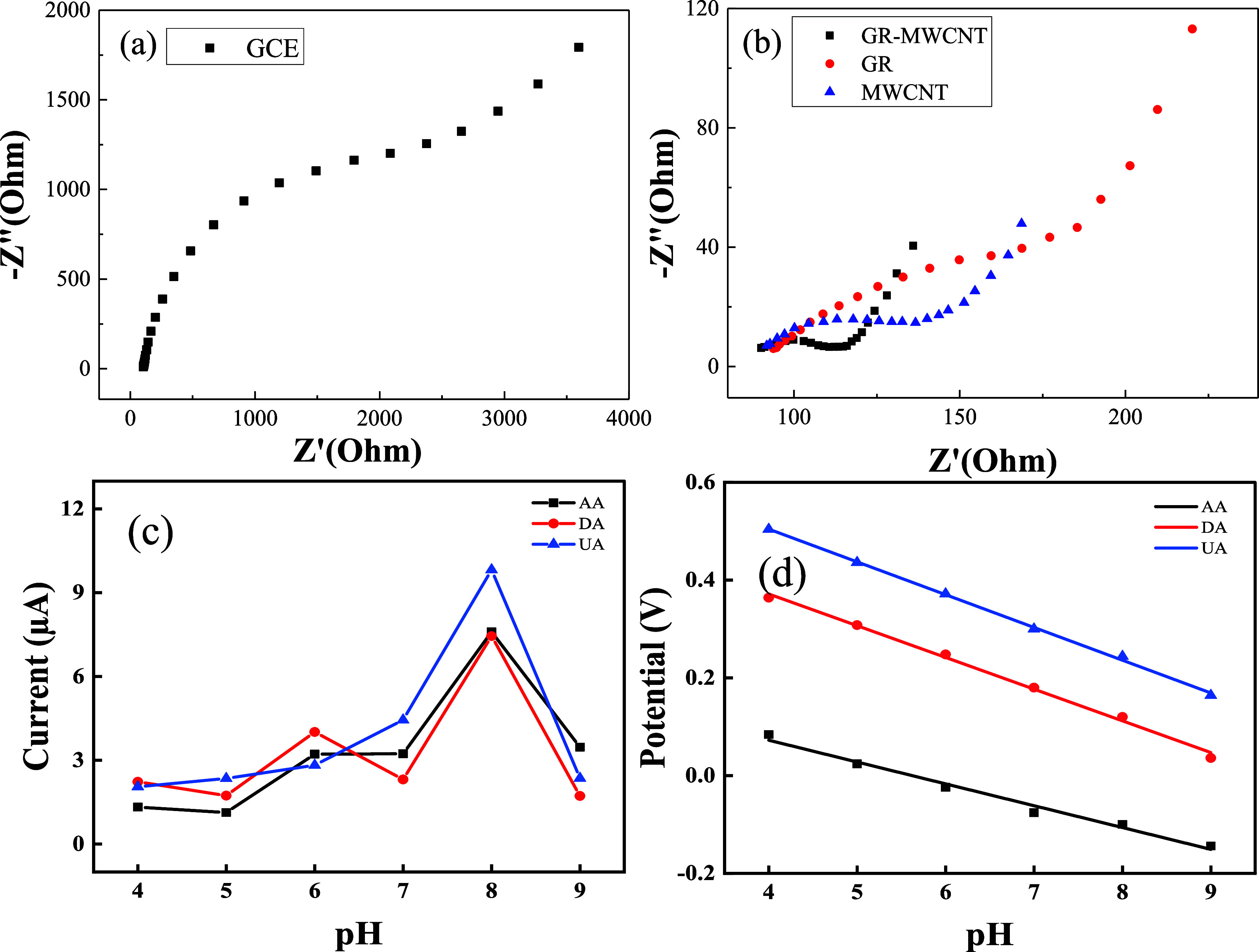
EIS spectra of (a) GCE, (b) GR/GCE, MWCNT/GCE, and GR-MWCNT/GCE in 0.1 M KCl containing 5 mM K_3_[Fe(CN)_6_]; influence of pH (4–9) on (c) peak current and (d) peak potentials obtained from DPVs for 100 μM AA, 5 μM DA, and 50 μM UA at GR-MWCNT/GCE.

Electrochemical impedance spectroscopy (EIS) was employed as an effective tool to investigate the interfacial properties of the electrode surfaces. Figure 4a,b presents the EIS spectra of GCE, GR/GCE, MWCNT/GCE, and GR-MWCNT/GCE in 0.1 M KCl containing 5 mM K_3_[Fe(CN)_6_] over a frequency range of 10 MHz–100 kHz. A typical Nyquist plot comprises a semicircular region, representing an electron transfer-limited process, and a linear region, indicative of a diffusion-limited process. Based on the Nyquist plots, the semicircular diameter correlates with the surface electron transfer resistance (*R*_ct_). The *R*_ct_ values for GCE, GR/GCE, MWCNT/GCE, and GR-MWCNT/GCE were 3492, 253, 154, and 92 Ω, respectively. These results demonstrate a significant enhancement in conductivity for the GR-MWCNT composite. This improvement highlights the superior electrical conductivity and large surface area of GR-MWCNT/GCE, facilitating efficient electron transfer between the target molecules and the electrode surface.

The pH value of the analytical solution is a critical factor in electrochemical studies, as protons participate directly in electrode reactions. To investigate this, the effect of pH on the oxidation current and potential of GR-MWCNT/GCE was examined using solutions containing 100 μM AA, 5 μM DA, and 50 μM UA. The results, shown in Figure 4c,d, indicate that the largest peak current appeared at the pH value of 8. It also demonstrates that the solution’s acidity significantly influences the oxidation peak potentials of AA, DA, and UA. As the pH increased, the oxidation potentials of all three analytes shifted negatively. The corresponding linear regression equations for the oxidation peak potentials (*E*_pa_) were as follows:
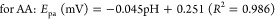

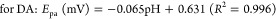

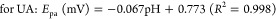
These results indicate that the electrochemical oxidation of AA, DA, and UA follows the Nernst equation, confirming that the oxidation processes for all three analytes involve two-proton, two-electron transfer mechanisms. Considering that the physiological pH of the human body is 7, all subsequent tests were conducted at pH 7.

### 3.4 CVs for Simultaneous Determination of AA, DA, and UA

**Figure 5:**
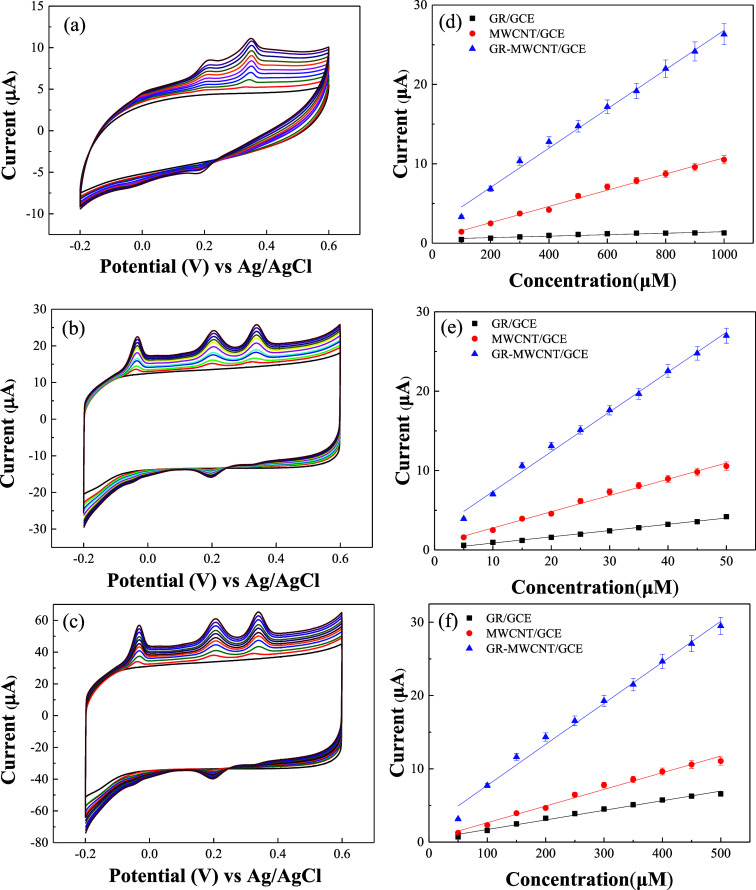
CVs of (a) GR/GCE, (b) MWCNT/GCE, and (c) GR-MWCNT/GCE in 0.1 M PBS containing various concentrations of AA (100–1000 μM), DA (5–50 μM), and UA (50–500 μM); calibration curves for (d) AA, (e) DA, and (f) UA on GR/GCE, MWCNT/GCE, and GR-MWCNT/GCE.

Figure 5a–c shows the CVs of GR/GCE, MWCNT/GCE, and GR-MWCNT/GCE, respectively, in a 0.1 M PBS solution for the simultaneous detection of different concentrations of UA (100–1000 μM), DA (5–50 μM), and UA (50–500 μM). Figure 5d–f illustrates the calibration curves of peak currents corresponding to the oxidation of AA, DA, and UA at different concentrations for the GR/GCE, MWCNT/GCE, and GR-MWCNT/GCE, respectively. The GR/GCE, MWCNT/GCE, and GR-MWCNT/GCE successfully detected AA, DA, and UA, showing a good linear relationship between the oxidation peak current and the concentration of the analytes. The slope of the calibration curve represents the sensitivity. Table 1 compares the oxidation onset potentials and sensitivities of the three electrodes for AA, DA, and UA. It can be observed that MWCNT/GCE and GR-MWCNT/GCE exhibit lower overpotentials for the detection of AA, DA, and UA, while GR-MWCNT/GCE shows the highest sensitivity. The sensitivity of the GR-MWCNT/GCE for AA, DA, and UA is 25, 3, and 3 times higher than that of the MWCNT/GCE and 25, 6, and 5 times higher than that of the GR/GCE, respectively.

**Table 1:** Onset Potential and Sensitivity of GR/GCE, MWCNT/GCE, and GR-MWCNT/GCE for Simultaneous Determination of AA, DA, and UA

electrode	analyte	onset potential (V vs Ag/AgCl)	sensitivity (μA μM^–1^)
GR/GCE	AA	–0.001	0.001
	DA	0.208	0.079
	UA	0.349	0.013
MWCNT/GCE	AA	–0.032	0.001
	DA	0.206	0.20
	UA	0.340	0.022
GR-MWCNT/GCE	AA	–0.032	0.025
	DA	0.206	0.50
	UA	0.340	0.059

**Figure 6:**
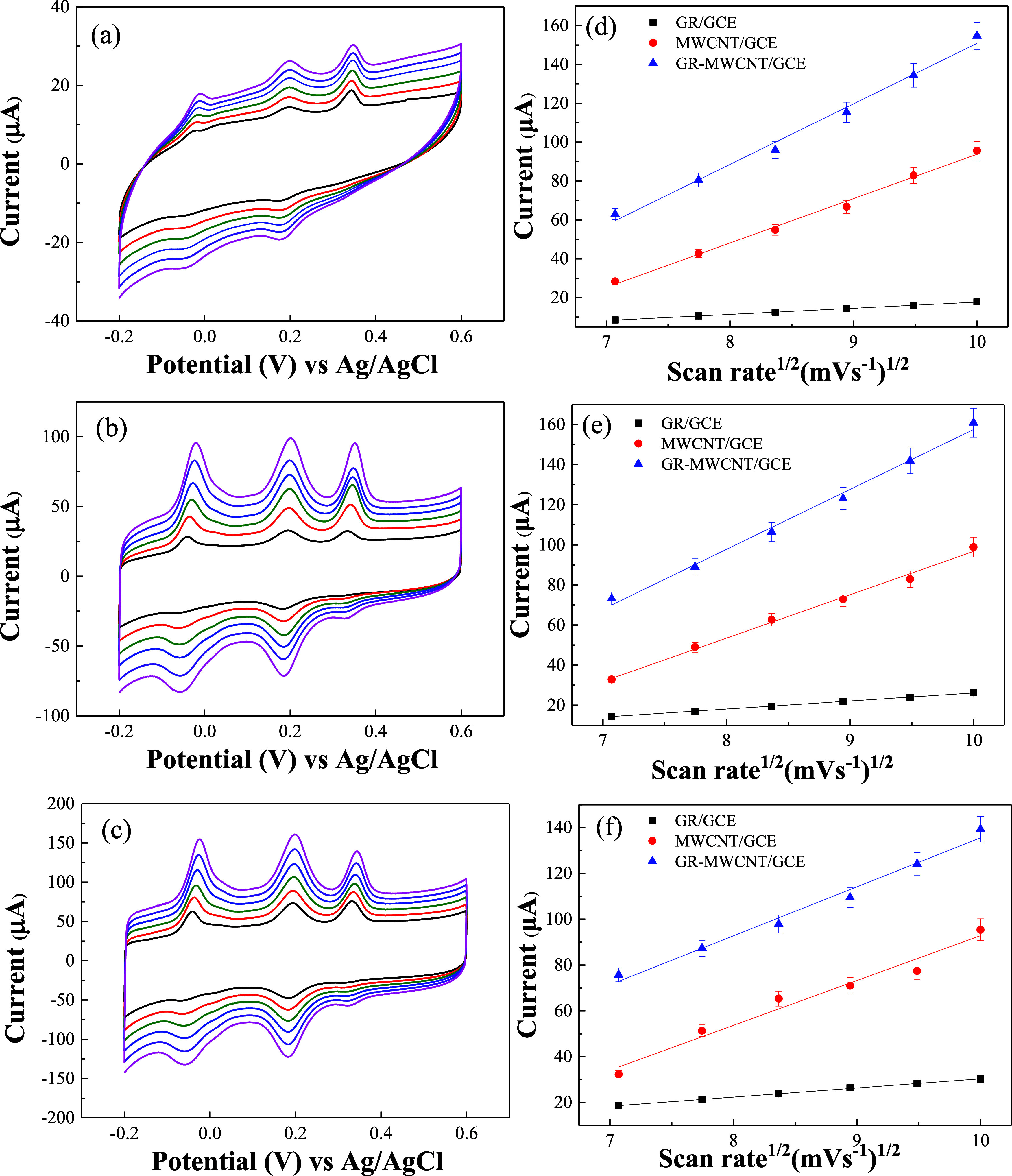
CVs of (a) GR/GCE, (b) MWCNT/GCE, and (c) GR-MWCNT/GCE in 0.1 M PBS containing 1000 μM AA, 50 μM DA, and 500 μM UA with scan rates from 50 to 100 mV/s. The linear relationship between the oxidation peak current and the square root of the scan rate for (d) AA, (e) DA, and (f) UA on GR/GCE, MWCNT/GCE, and GR-MWCNT/GCE.

The electrochemical behavior of simultaneously detecting AA, DA, and UA using GR/GCE, MWCNT/GCE, and GR-MWCNT/GCE was analyzed by varying the scan rates and examining the relationship between the oxidation peak current and the square root of the scan rate. Figure 6a–c shows the CVs obtained for GR/GCE, MWCNT/GCE, and GR-MWCNT/GCE, respectively, in the presence of 1000 μM AA, 50 μM DA, and 500 μM UA, with scan rates ranging from 50 to 100 mV/s. Figure 6d–f illustrates the plots of the peak currents for the oxidation of AA, DA, and UA against the square root of the scan rate. The oxidation peak currents for AA, DA, and UA increase significantly with the increase in scan rate, and the oxidation peak potentials of the three electrodes shift slightly to more positive potentials with an increasing scan rate. Additionally, the oxidation peak currents of the three electrodes exhibit a good linear relationship with the square root of the scan rate, indicating that the electrooxidation reactions of AA, DA, and UA at the three electrodes are diffusion-controlled. Regardless of the scan rate, the GR-MWCNT/GCE electrode provided the most distinct oxidation signals for the simultaneous detection of AA, DA, and UA.

### 3.5 DPVs for the Simultaneous Determination of AA, DA, and UA

**Figure 7:**
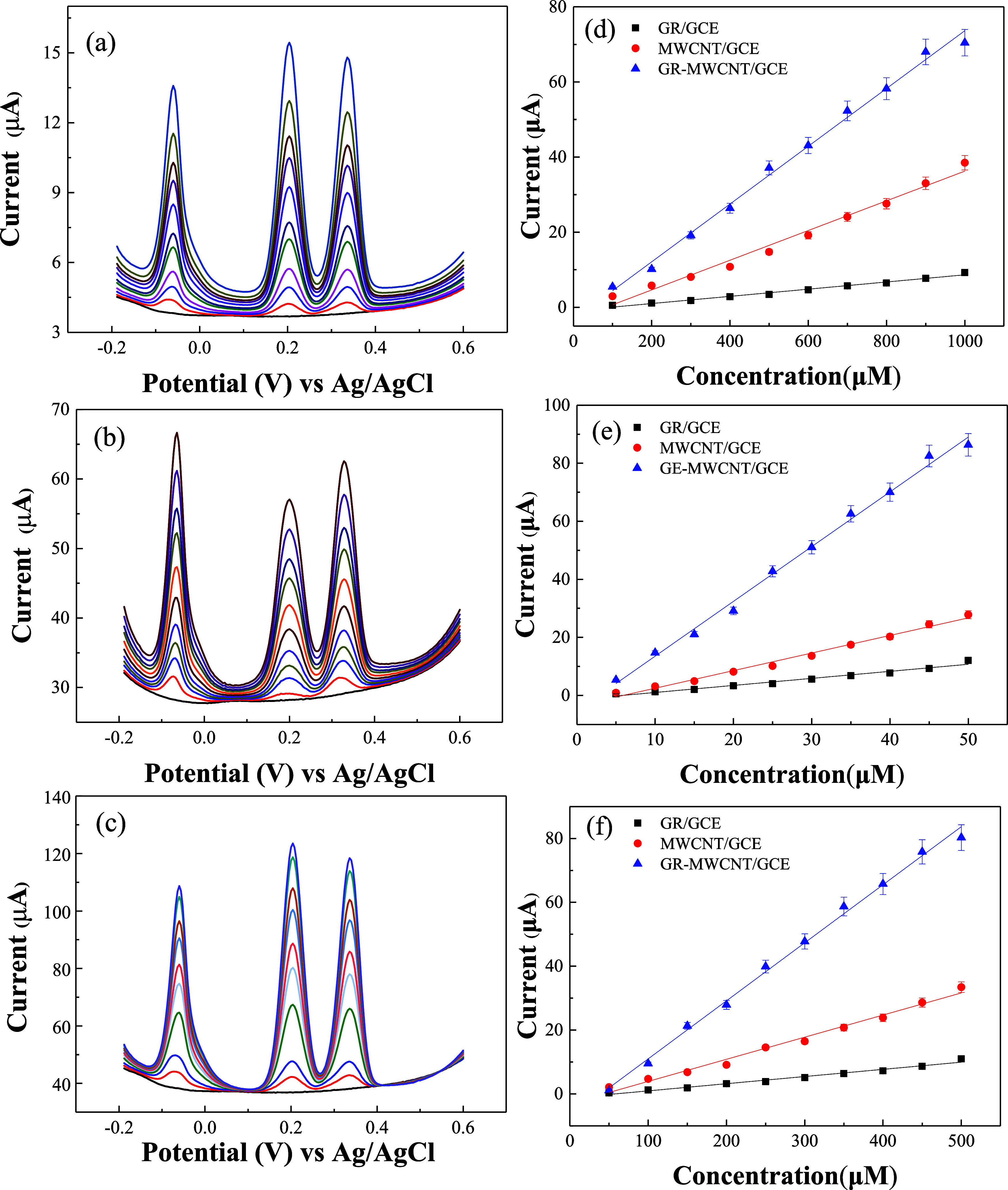
DPVs of (a) GR/GCE, (b) MWCNT/GCE, and (c) GR-MWCNT/GCE in 0.1 M PBS containing various concentrations of AA (100–1000 μM), DA (5–50 μM), and UA (50–500 μM). The linear relationship between the peak current and concentration of (d) AA, (e) DA, and (f) UA on GR/GCE, MWCNT/GCE, and GR-MWCNT/GCE.

Figure 7a–c shows the DPVs of GR/GCE, MWCNT/GCE, and GR-MWCNT/GCE, respectively, for the simultaneous detection of AA, DA, and UA in a 0.1 M PBS solution. The concentration ranges for AA, DA, and UA were set at 100–1000, 5–50, and 50–500 μM, respectively. All three electrodes demonstrated the capability of detecting AA, DA, and UA simultaneously without interference from one another. As the concentrations of AA, DA, and UA increased, their corresponding peak currents also increased. The plots of peak current versus concentration for the detection of AA, DA, and UA using GR/GCE, MWCNT/GCE, and GR-MWCNT/GCE exhibited good linear relationships, as shown in Figure 7d–f. We also conducted individual DPVs of AA, DA, and UA using GR/GCE, MWCNT/GCE, and GR-MWCNT/GCE, respectively. The results are presented in Figures S1–S3, with the summarized data provided in Table S1. It was observed that the overpotentials for DA and UA detection using GR/GCE, MWCNT/GCE, and GR-MWCNT/GCE were approximately 0.05 V versus Ag/AgCl lower in individual detection compared to simultaneous detection. However, the sensitivity was found to be very similar, regardless of whether AA, DA, and UA were detected simultaneously or individually.

To investigate the detection limits (LOD) of GR-MWCNT/GCE, we further reduced the analyte concentrations to AA (10–100 μM), DA (1–10 μM), and UA (5–50 μM), as shown in Figure S4. The LOD was determined using the definitions of the International Union of Pure and Applied Chemistry (IUPAC). Table 2 compares the oxidation potential, sensitivity, and LOD of GR/GCE, MWCNT/GCE, and GR-MWCNT/GCE for the detection of AA, DA, and UA simultaneously. It can be observed that GR-MWCNT/GCE exhibited the highest sensitivity and the lowest detection limit for the detection of AA (6.71 μM), DA (0.58 μM), and UA (7.30 μM). The sensitivity of GR-MWCNT/GCE for the simultaneous detection of AA, DA, and UA using DPV is 0.076, 1.38, and 0.18 μA μM^–1^, respectively, which is 3, 2.8, and 3 times higher than that detected using CV. The high sensitivity of the GR-MWCNT/GCE is attributed to the interactions within the composite material, which introduce more defects and thereby increase the active surface area available for sensing the analytes.

**Table 2:** Comparison of the Oxidation Potential, Sensitivity, and Detection Limit of GR/GCE, MWCNT/GCE, and GR-MWCNT/GCE for the Simultaneous Detection of AA, DA, and UA

electrode	analyte	oxidation potential (V vs Ag/AgCl)	sensitivity (μA μM^–1^)	LOD (μM)	*R*^2^
GR/GCE	AA	–0.06	0.009	83.0	0.985
	DA	0.204	0.24	6.04	0.969
	UA	0.336	0.023	51.02	0.976
MWCNT/GCE	AA	–0.064	0.039	91.72	0.982
	DA	0.2	0.606	3.72	0.988
	UA	0.328	0.070	41.39	0.985
GR/MWCNT/GCE	AA	–0.06	0.076	6.71	0.993
	DA	0.204	1.38	0.58	0.994
	UA	0.336	0.181	7.30	0.995

**Figure 8:**
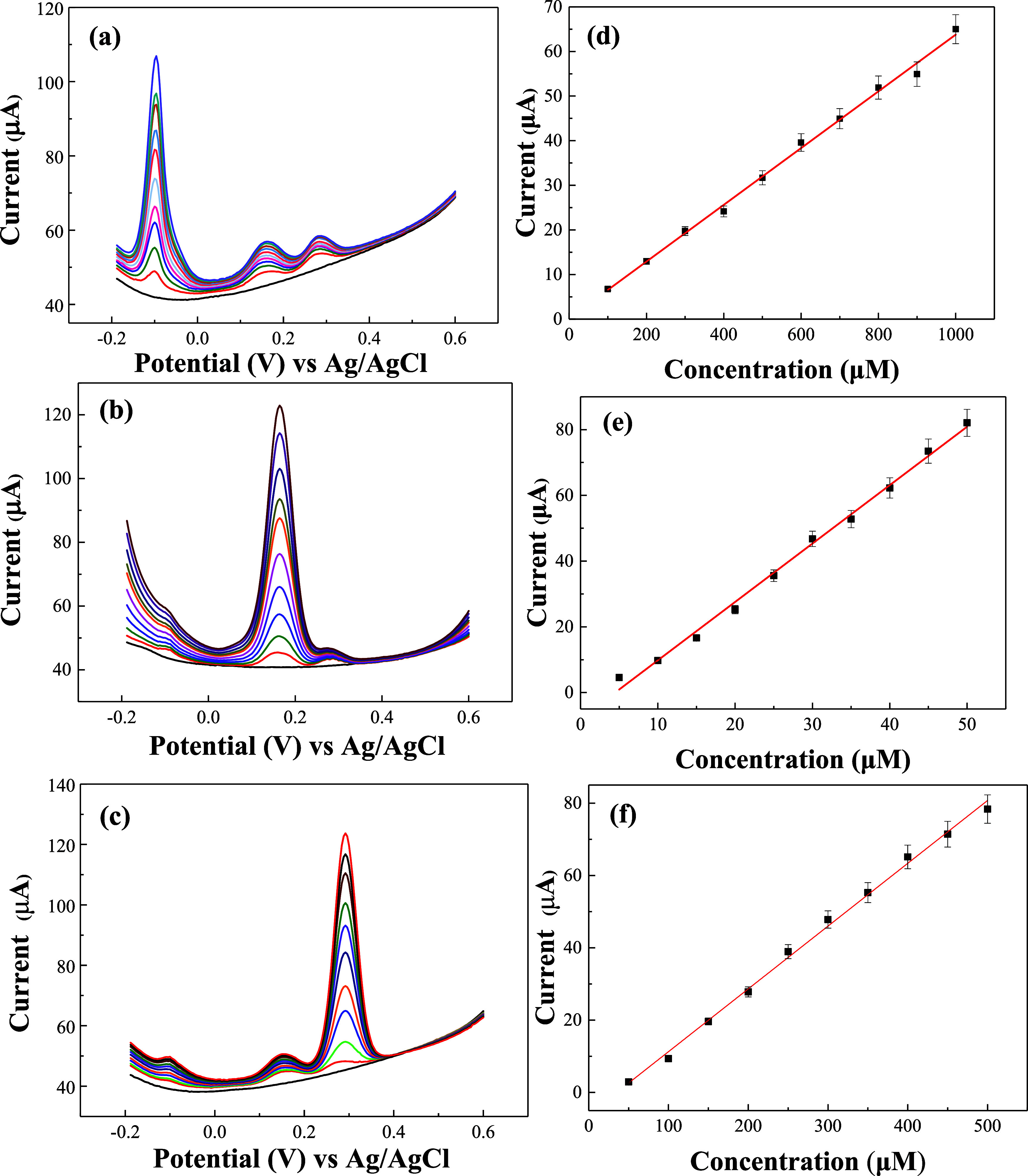
DPVs of GR-MWCNT/GCE in 0.1 M PBS containing (a) 5 μM DA, 50 μM UA, and different concentrations of AA from 100 to 1000 μM; (b) 100 μM AA, 50 μM UA, and different concentrations of DA from 5 to 50 μM; (c) 100 μM AA, 5 μM DA, and different concentrations of UA from 50 to 500 μM. The linear relationship between peak current and concentration of (d) AA, (e) DA, and (f) UA.

On the other hand, for the GR-MWCNT/GCE, we also tested the cross-interference among AA, DA, and UA. As shown in Figure 8, DPVs were employed to investigate the simultaneous detection of AA, DA, and UA by varying the concentration of one analyte while keeping the other two constant. The results indicate that the peak currents of AA, DA, and UA are proportional to their respective concentrations with minimal influence from the other two analytes. This suggests that the responses of AA, DA, and UA at the GR-MWCNT/GCE are relatively independent. The sensitivities for AA, DA, and UA were determined to be 0.066, 1.70, and 0.174 μA μM^–1^, respectively, with calibration ranges of 100–1000, 5–50, and 50–500 μM. These sensitivities are comparable to those obtained when the concentrations of all three analytes were varied simultaneously. These findings confirm the feasibility of simultaneous detection of UA, AA, and DA in a coexisting system using the GR-MWCNT/GCE.

### 3.6 Reproducibility, Stability, and Interferences

**Figure 9:**
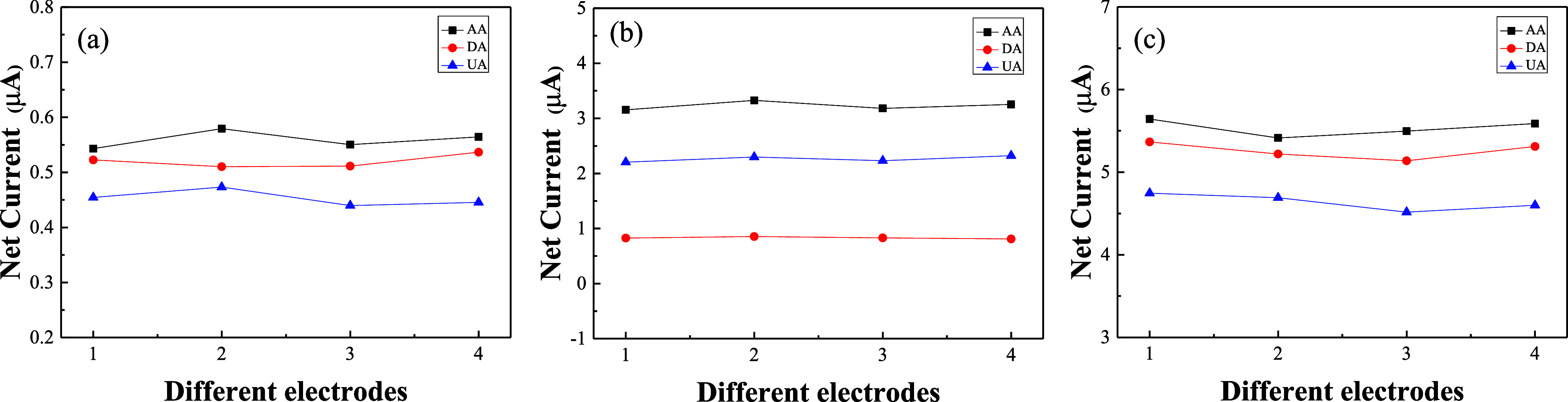
Net current of (a) GR/GCE, (b) MWCNT/GCE, and (c) GR-MWCNT/GCE in 0.1 M PBS containing 100 μM AA, 5 μM DA, and 50 μM UA for four independent electrodes.

To verify the reproducibility of the preparation of GR/GCE, MWCNT/GCE, and GR-MWCNT/GCE, four sets of modified electrodes were independently fabricated based on the same bare electrode and then tested in the presence of a mixture of 100 μM AA, 5 μM DA, and 50 μM UA, as shown in Figure 9a–c. All three electrodes exhibited good reproducibility during preparation. Among them, GR-MWCNT/GCE demonstrates the lowest relative standard deviation (RSD) for AA, DA, and UA, with values of 1.83, 1.92, and 2.17, respectively.

**Figure 10:**
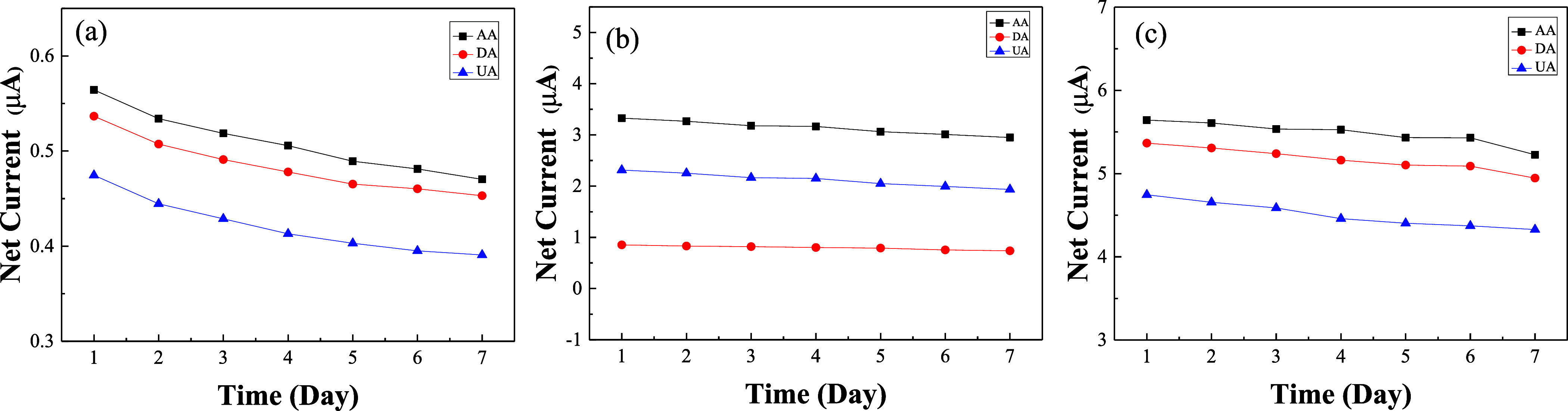
Net current of (a) GR/GCE, (b) MWCNT/GCE, and (c) GR-MWCNT/GCE containing 100 μM AA, 5 μM DA, and 50 μM UA at different days.

The stability of GR/GCE, MWCNT/GCE, and GR-MWCNT/GCE was examined by storing the electrodes in air at room temperature. The electrodes were tested once daily for seven consecutive days, as shown in Figure 10a–c. All three electrodes consistently separated the oxidation peak currents of 100 μM AA, 5 μM DA, and 50 μM UA in the mixture on each day. By the seventh day, the GR-MWCNT/GCE maintained the highest peak current signals for AA, DA, and UA, retaining 93, 92, and 91% of the initial measurements, as shown in Figure 10c, indicating that the GR-MWCNT/GCE possesses good stability. Table 3 compares the reproducibility and stability of GR/GCE, MWCNT/GCE, and GR-MWCNT/GCE. The results indicate that GR-MWCNT/GCE exhibits the best reproducibility and stability among the three.

**Table 3:** Reproducibility and Stability of GR/GCE, MWCNT/GCE, and GR-MWCNT/GCE in the Presence of a Mixture of 100 μM AA, 5 μM DA, and 50 μM UA

electrode	analyte	reproducibility RSD (%)	stability (%)
GR/GCE	AA	2.86	83
	DA	2.35	85
	UA	3.24	82
MWCNT/GCE	AA	2.4	89
	DA	2.2	87
	UA	2.44	84
GR-MWCNT/GCE	AA	1.83	93
	DA	1.92	92
	UA	2.17	91

**Figure 11:**
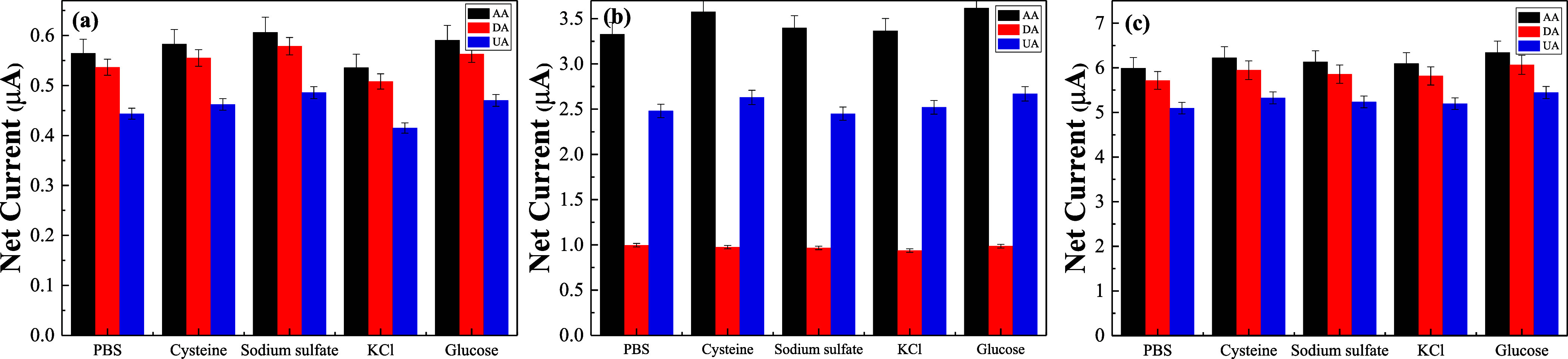
Interference study of some foreign substances in the presence of 100 μM AA, 5 μM DA, and 50 μM UA in PBS by DPV at (a) GR/GCE, (b) MWCNT/GCE, and (c) GR-MWCNT/GCE.

The selectivity of GR/GCE, MWCNT/GCE, and GR-MWCNT/GCE was evaluated in a 0.1 M PBS solution containing 100 μM AA, 5 μM DA, and 50 μM UA. Potential interfering substances, including 1 mM cysteine, sodium sulfate, potassium chloride, and glucose, were introduced to assess the selectivity. Figure 11a–c shows the net current values of AA, DA, and UA obtained from GR/GCE, MWCNT/GCE, and GR-MWCNT/GCE in the presence of these interfering substances. The results indicate that the interferents had a minimal impact on the simultaneous detection of AA, DA, and UA by all three electrodes. The RSD for AA, DA, and UA were 4.7, 4.96, and 4.97% for GR/GCE; 3.78, 2.36, and 3.75% for MWCNT/GCE; and 2.16, 2.26, and 2.53% for GR-MWCNT/GCE, respectively. These results demonstrate that the GR-MWCNT/GCE electrode exhibits the best anti-interference capability among the three modified electrodes.

### 3.7 Analysis of AA, DA, and UA in Real Biological Samples

To evaluate the sensing performance of the GR-MWCNT/GCE in real samples, tests were conducted using serum and urine samples. The blood samples were first centrifuged to obtain the serum, which was then diluted 100 times with a 0.1 M PBS solution. Different concentrations of the three analytes were added to the serum samples, and DPV analysis was performed by using the standard addition method to compare the added concentrations with the actual detected concentrations. The experimental results are shown in Table 4, where the recovery rates for AA, DA, and UA in the spiked serum samples ranged from 98.6 to 103.7%, 98.4 to 103.8%, and 95.92 to 103.76%, respectively, indicating that GR-MWCNT/GCE performed well in detecting AA, DA, and UA in serum samples.

**Table 4:** Determination of AA, DA, and UA in Serum and Urine Samples using GR-MWCNT/GCE and DPV in 0.1 M PBS

sample	analyte	spiking (μM)	found (μM)	recovery (%)
serum	AA	100	103.7	103.7
	AA	500	499.87	99.7
	AA	1000	986.01	98.6
	DA	5	5.08	101.6
	DA	25	24.6	98.4
	DA	50	51.9	103.8
	UA	50	51.88	103.76
	UA	250	239.81	95.92
	UA	500	500.66	100.13
urine	AA	100	91.01	91.01
	AA	500	479	95.8
	AA	1000	990.2	99.02
	DA	5	4.85	97
	DA	25	25.21	100.84
	DA	50	50.4	100.8
	UA	50	52.14	104.28
	UA	250	259.28	103.71
	UA	500	521.27	104.25

For urine samples, a 50-fold dilution was performed using a 0.1 M PBS solution before spiking with different concentrations of the three analytes. DPV analysis was also performed using the standard addition method, with the results presented in Table 4. The recovery rates for AA, DA, and UA in the spiked urine samples were found to be 91.01 to 99.02%, 97 to 100.84%, and 103.71 to 104.28%, respectively, demonstrating that GR-MWCNT/GCE also exhibited good performance in detecting AA, DA, and UA in urine samples.

## 4 Conclusions

In this study, the sensing performance of GR/GCE, MWCNT/GCE, and GR-MWCNT/GCE for the simultaneous detection of AA, DA, and UA was compared using CV and DPV. All three modified electrodes were capable of simultaneously detecting AA, DA, and UA, with GR-MWCNT/GCE showing the highest sensitivity. The sensing current of the GR-MWCNT/GCE exhibited a good linear relationship with the concentration of the analytes. The sensitivity of GR-MWCNT/GCE for detecting AA (100–1000 μM), DA (5–50 μM), and UA (50–500 μM) was 0.076, 1.38, and 0.181 μA μM^–1^, respectively, with detection limits of 6.71, 0.58, and 7.30 μM, respectively. The electrode also demonstrated good stability, reproducibility, and excellent anti-interference capability. Furthermore, it was able to accurately and sensitively detect AA, DA, and UA in real samples with high recovery rates. In serum samples, the recovery rates for AA, DA, and UA were 98.6–103.7%, 98.4–103.8%, and 95.92–103.76%, respectively, while in urine samples, the recovery rates were 91.01–99.02%, 97–100.84%, and 103.71–104.28%, respectively. These superior performances are attributed to the synergistic effects between GR and the MWCNT, which enhance the defect density and electroactive surface area of the electrode while reducing surface resistance. This enables efficient catalytic reactions involving two-proton, two-electron transfer processes, allowing for the successful detection of AA, DA, and UA.

## Supporting Information Available

DPVs on GR/GCE, MWCNT/GCE, and GR-MWCNT/GCE in 0.1 M PBS containing various AA concentrations from 100 to 1000 μM; calibration curves for AA on GR/GCE, MWCNT/GCE, and GR-MWCNT/GCE; DPVs on GR/GCE, MWCNT/GCE, and GR-MWCNT/GCE in 0.1 M PBS containing various DA concentrations from 5 to 50 μM; calibration curves for DA on GR/GCE, MWCNT/GCE, and GR-MWCNT/GCE; DPVs on GR/GCE, MWCNT/GCE, and GR-MWCNT/GCE in 0.1 M PBS containing various UA concentrations from 50 to 500 μM; calibration curves for UA on GR/GCE, MWCNT/GCE, and GR-MWCNT/GCE; a comparison of the oxidation potential, sensitivity, and detection limit of GR/GCE, MWCNT/GCE, and GR-MWCNT/GCE for the individual detection of AA, DA, and UA; and DPVs of GR-MWCNT/GCE in 0.1 M PBS containing various concentrations of AA (0–100 μM), DA (0–10 μM), and UA (0–50 μM) (PDF)
