# Supplementary material for: Graphene–Multiwalled Carbon Nanotubes Modified Glassy Carbon Electrodes for Simultaneous Detection of Ascorbic Acid, Dopamine, and Uric Acid
Source: ACS Omega. 2025 Feb 20;10(8):8160–71. doi: 10.1021/acsomega.4c09646 (PMC11886922; doi:10.1021/acsomega.4c09646)
Supplement: Supplementary file 1 — ao4c09646_si_001.pdf [file ao4c09646_si_001.pdf]

Graphene-Multi-Walled Carbon Nanotubes Modified Glassy Carbon Electrode for Simultaneous Detection of Ascorbic Acid, Dopamine, and Uric Acid

Hsien-Hsu Hsieh, Jie-Yu Xu, Jing-Tong Lin, Yun-Ting Chiang and Yu-Ching Weng*

Department of Chemical Engineering, Feng Chia University*, Taichung, Taiwan*

*Corresponding author.

Tel.: 886-4-24517250 ext. 3689; Fax: 886-4-24510890; Email: ycweng@fcu.edu.tw

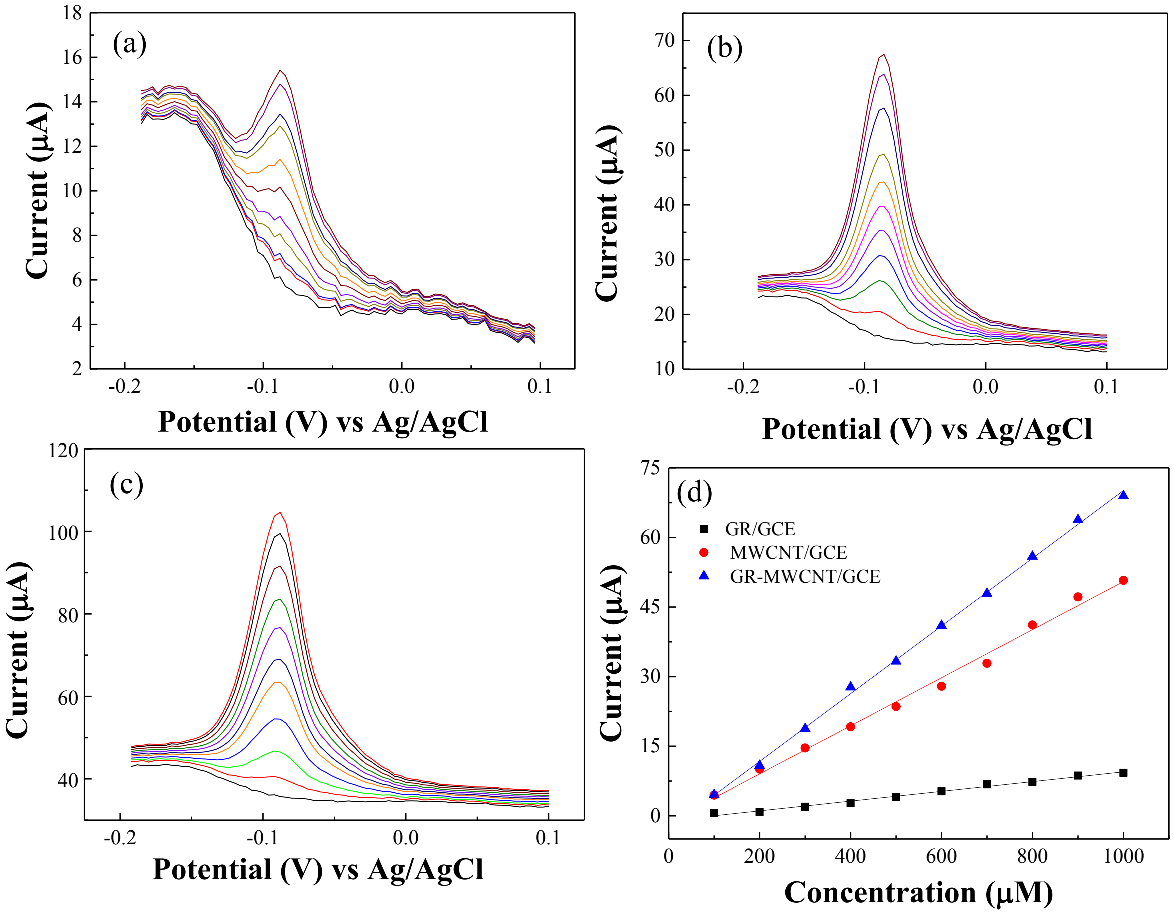

Fig. S1 DPVs on (a) GR/GCE, (b) MWCNT/GCE, and (c) GR-MWCNT/GCE in 0.1 M PBS containing various AA concentrations from 100 to 1000 μM; (d) Calibration curves for AA on GR/GCE, MWCNT/GCE, and GR-MWCNT/GCE.

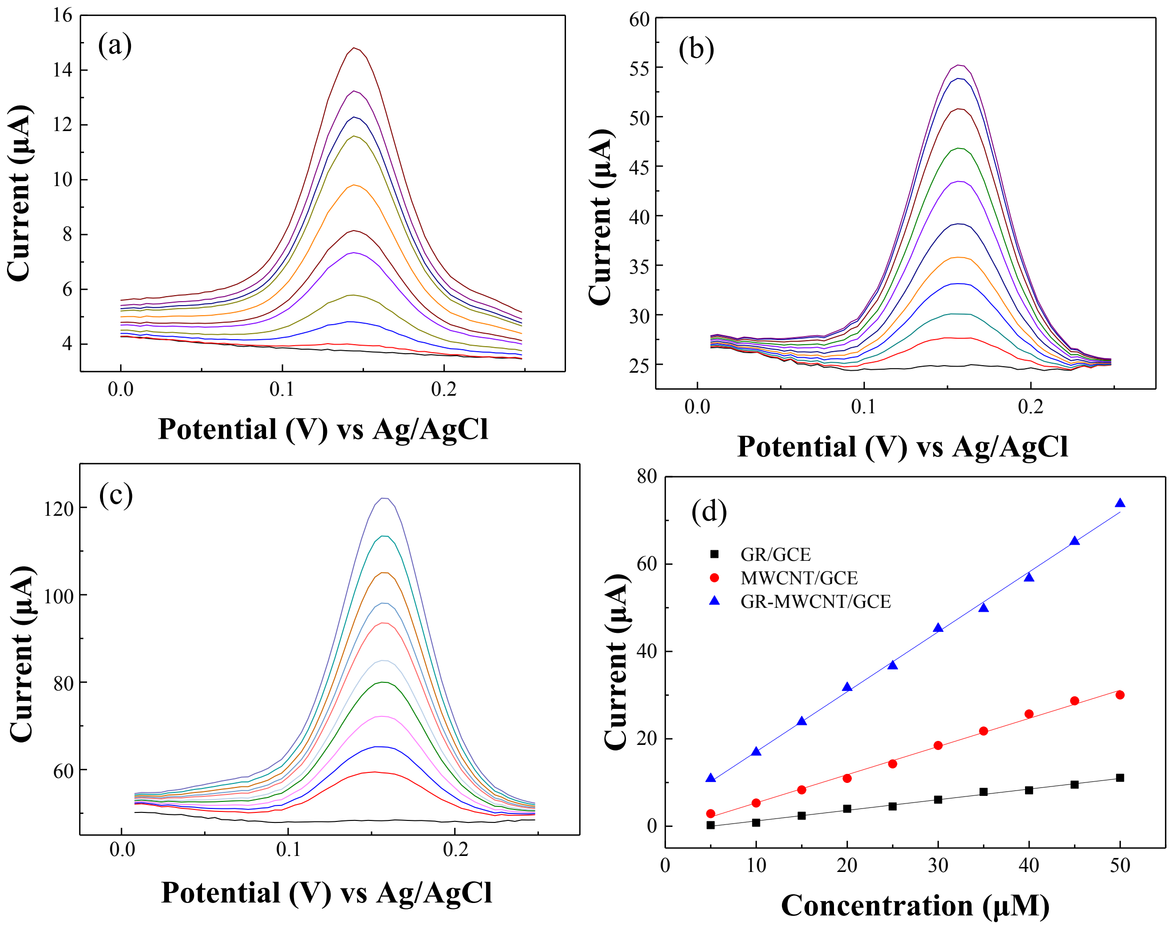

Fig. S2 DPVs on (a) GR/GCE, (b) MWCNT/GCE, and (c) GR-MWCNT/GCE in 0.1 M PBS containing various DA concentrations from 5 to 50 μM; (d) Calibration curves for DA on GR/GCE, MWCNT/GCE, and GR-MWCNT/GCE.

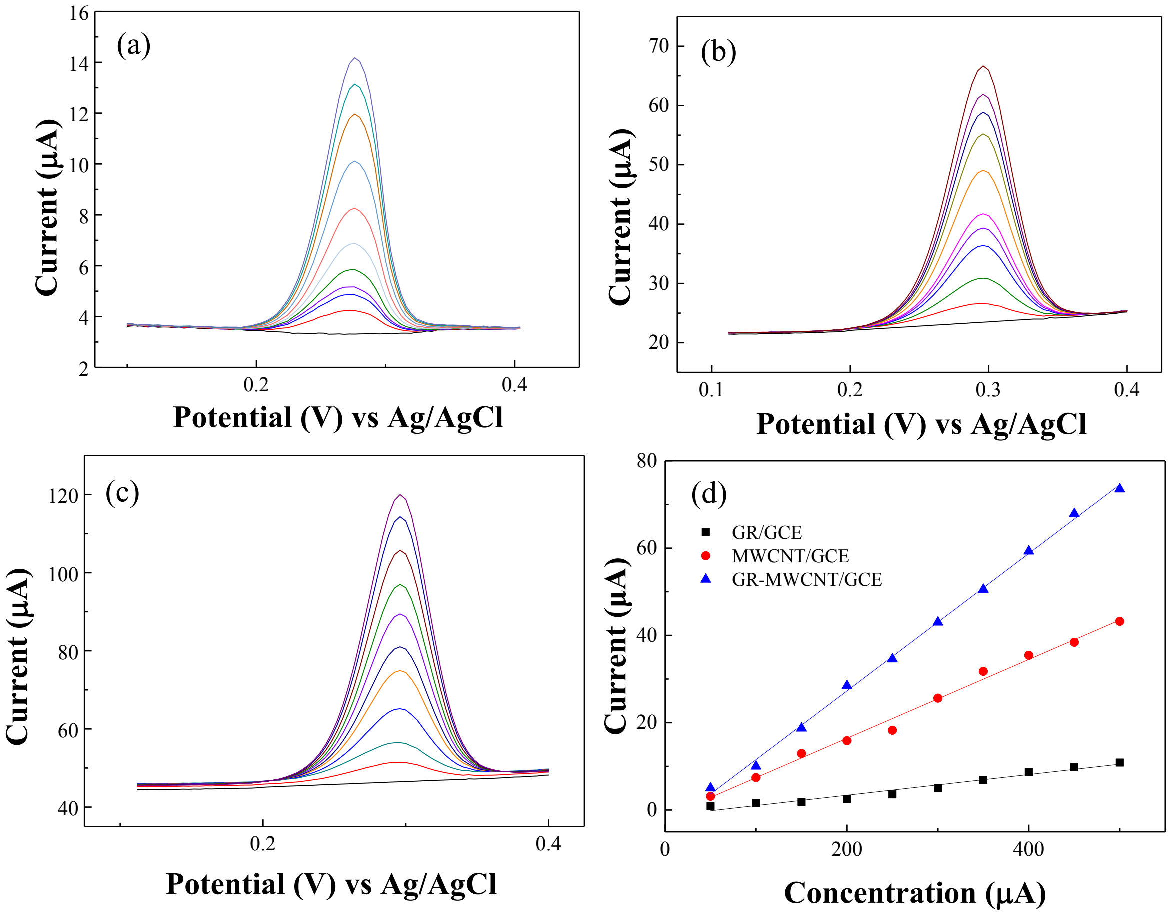

Fig. S3 DPVs on (a) GR/GCE, (b) MWCNT/GCE, and (c) GR-MWCNT/GCE in 0.1 M PBS containing various UA concentrations from 50 to 500 μM; (d) Calibration curves for UA on GR/GCE, MWCNT/GCE, and GR-MWCNT/GCE.

Table S1 Comparison of the oxidation potential, sensitivity, and detection limit of GR/GCE, MWCNT/GCE, and GR-MWCNT/GCE for the individual detection of AA, DA, and UA.

Electrode	Analyte	Oxidation potential (V vs Ag/AgCl)	Sensitivity (μAμM^{-1})	LOD (μM)	R^2
GR/GCE	AA	-0.088	0.007	90.4	0.978
	DA	0.144	0.24	3.22	0.981
	UA	0.276	0.023	72.6	0.956
MWCNT/GCE	AA	-0.084	0.052	58.48	0.992
	DA	0.152	0.65	2.75	0.993
	UA	0.296	0.09	31.89	0.991
GR/MWCNT/GCE	AA	-0.088	0.073	25.6	0.998
	DA	0.156	1.37	1.91	0.997
	UA	0.300	0.157	15.4	0.997

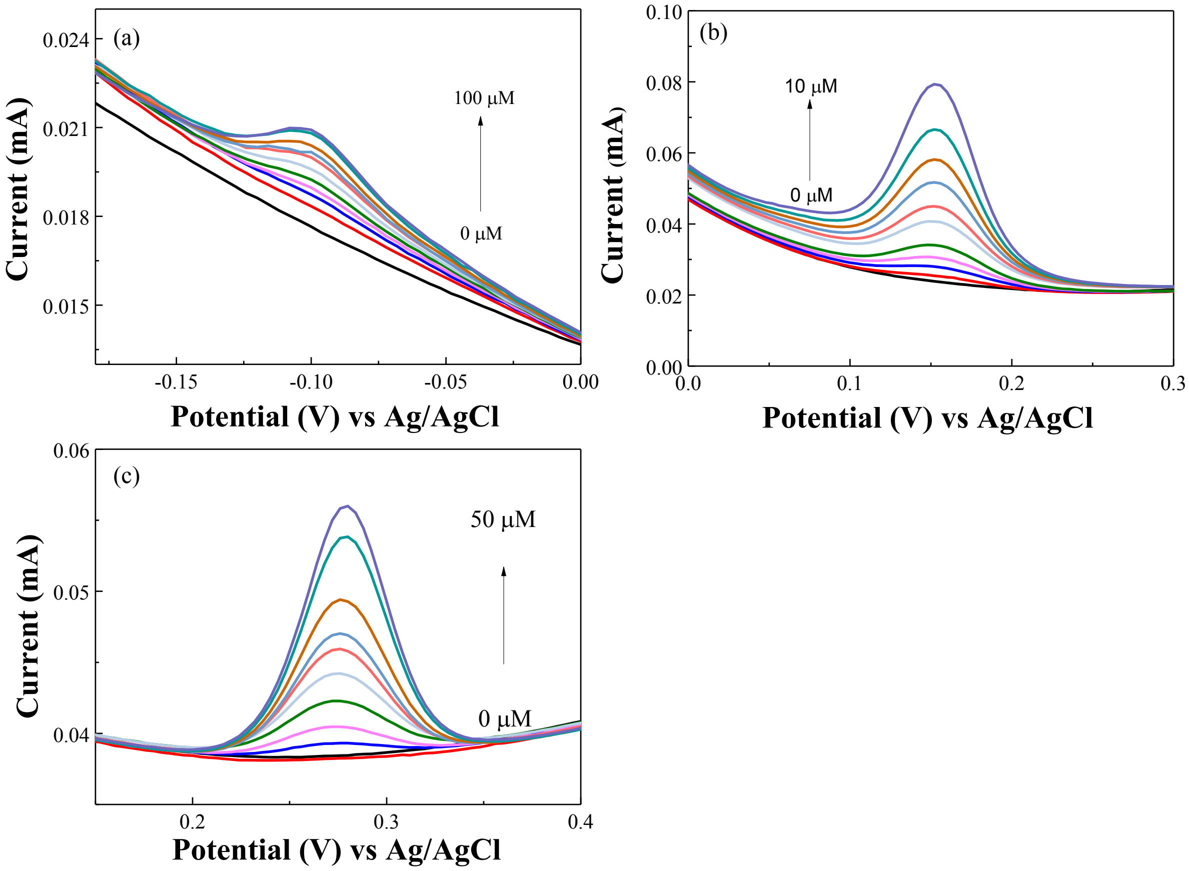

Fig. S4 DPVs of GR-MWCNT/GCE in 0.1 M PBS containing various concentrations of (a) AA (0-100 μM), (b) DA (0-10 μM), and UA (0-50 μM).
